# Intradermal injection of human adipose-derived stem cells accelerates skin wound healing in nude mice

**DOI:** 10.1186/s13287-015-0238-3

**Published:** 2015-12-08

**Authors:** Jonathan Rodriguez, Fabien Boucher, Charlotte Lequeux, Audrey Josset-Lamaugarny, Ondine Rouyer, Orianne Ardisson, Héléna Rutschi, Dominique Sigaudo-Roussel, Odile Damour, Ali Mojallal

**Affiliations:** Banque de tissus et cellules, Laboratoire des substituts cutanés, Hôpital Edouard Herriot, Hospices Civils de Lyon, 5, place d’Arsonval, Pavillon i, 69437 Lyon, France; INSERM U1060, CarMeN laboratory, Oullins, France; Service de chirurgie plastique, esthétique et reconstructrice, Hospices Civils de Lyon, University of Lyon, Lyon, France; IBCP-UMR 5305 CNRS, 7 passage du Vercors, 69 367 Lyon, Cedex 07 France; Laboratoire Central d’Anatomie Pathologique, Hôpital Édouard Herriot, Lyon, France; Cell and Tissue Bank, Cutaneous Substitute Laboratory, Edouard Herriot Hospital, 5, place d’Arsonval, Pavillon I, 69437 Lyon, France

**Keywords:** Adipose-derived stem cells, Cutaneous wound healing, Skin blood perfusion, Vehicle

## Abstract

**Background:**

The use of stem cells from adipose tissue or adipose-derived stem cells (ASCs) in regenerative medicine could be an interesting alternative to bone marrow stem cells because they are easily accessible and available in large quantities. The aim of this study was to evaluate the potential effect of ASCs on the healing of 12 mm diameter-excisional wounds (around 110 mm^2^) in nude mice.

**Methods:**

Thirty nude mice underwent surgery to create one 12-mm excisional wound per mouse (spontaneous healing, n = 6; Cytocare® 532, n = 12; ASCs, n = 12). The Galiano wound model was chosen to avoid shrinkage and thus slow the spontaneous healing (SH) of mouse skin, making it closer to the physiology of human skin healing. Transparent dressings were used to enable daily healing time measurements to be taken. Immunohistochemistry, histological and blood perfusion analysis were carried out on the healed skin.

**Results:**

The in vivo results showed the effectiveness of using ASCs on reducing the time needed for complete healing to 21.2 days for SH, 17.4 days for vehicle alone (Cytocare® 532) and 14.6 days with the addition of ASCs (p < 0.001). Moreover, cutaneous perfusion of the healed wound was significantly improved in ASC-treated mice compared to SH group, as shown by laser Doppler flowmetry and the quantitation of blood vessels using immunohistochemistry of αsmooth muscle actin.

**Conclusions:**

The tolerance and efficacy of cryopreserved ASCs to accelerate the complete closure of the wound by increasing the maturation of the skin and its blood perfusion, *s*hows their therapeutic benefit in the wound healing context.

## Background

Wounds are challenges that are often encountered in plastic and reconstructive surgery [1]. Indeed, healing is compromised in many situations such as diabetes, chronic renal failure, and irradiation, but also with age [2]. So, faced with an aging population today, chronic wounds are a real public health problem [3, 4]. The main aims of treatment are the rapid closure of the wound to restore the barrier function of the skin and prevent infection, the suppression of pain, and functional recovery, allowing a rapid return to a normal social and professional life, and equally important, obtaining a satisfactory result from an aesthetic point of view.

Many strategies have been tried with varying success in the treatment of chronic wounds: the injection of growth factors [5–7], grafts of temporary skin substitutes (porcine xenograft, synthetic membranes, atelocollagen matrix, and allogenic substitutes) [7, 8], or permanent ones (epidermal substitutes and cultured dermis) [9, 10]. However, large wounds, under adverse local and systemic conditions (low vascularization, metabolic disease, etc), respond poorly to these treatments and frequently reopen.

Over the last decade, plastic surgery has been marked by the introduction of many reconstructive therapies based on the use of stem cells. These are undifferentiated cells with self-renewing properties that are able to divide and generate specialized cells, including skin cells [11]. From this point of view, the stromal vascular fraction (SVF) of adipose tissue has been shown to be very effective in experimental healing models. The suggested mechanism of action is the increased cell proliferation and vascularization, modulation of inflammation, and increased fibroblastic activity of adipose-derived stem cells (ASCs) present in the SVF [12]. ASCs share many properties with mesenchymal stem cells from bone marrow and cord blood, including their multipotency and, in particular, their potential to differentiate into endothelial cells and fibroblasts [11]. However, SVF is a heterogeneous cell suspension containing a small percentage (between 1 and 3 %) of ASCs [13, 14]. Today, using protocols for the isolation and in vitro expansion of ASCs, it is easily possible to obtain a homogeneous therapeutic product containing more than 90 % ASCs [15]. In addition, the ASC amplification phase requires their extraction from only a small volume of lipoaspirate, which is particularly advantageous in frail, elderly or diabetic patients. Finally, the possibility of freezing ASCs pending their therapeutic use, offers the opportunity to 1) perform all the necessary safety checks before releasing the therapy product and 2) repeat the treatment if necessary.

Of the preclinical trials performed so far using ASCs, most have been performed on rodents [16–28] and only a few on porcine [29–33] and rabbit [34, 35] models, probably due to the lack of availability, high cost, management difficulty and the strong shrinkage of the wound, due to the presence of dermal muscles. The ready availability of murine models, together with ease of management and sampling, may explain their frequent use in such trials. Monitoring of the healing is most often done bi-weekly at the same time as the dressings are changed, which prevents a daily assessment of wound closure. For these reasons, we chose the Galiano wound model [36] to avoid significant shrinkage observed on mice and thus slow the closure of their wound, making it closer to the physiology of human skin healing. In addition, a larger wound area than usual (12 mm instead of 6 mm in most protocols) was created and covered with a transparent dressing in order to carry out daily measurements of the surface of the wound and assess the total healing time more precisely.

In this study, we aimed to evaluate, in this optimized healing model, the effectiveness of injecting a controlled dose of cultured ASCs, using different criteria such as wound healing kinetics and skin perfusion. Furthermore, in order to avoid their dispersion in the subcutaneous tissue, ASCs were injected in an European Community-approved (EC labeled) hyaluronic acid gel with added vitamins and minerals (Cytocare® 532, Revitacare Laboratory, Saint-Ouen-l’Aumône, France) that we tested for biocompatibility, bioavailability and tolerance [37].

## Methods

### Adipose tissue collection

Surgical residue was harvested according to French regulations and declared to the Research Ministry (DC n°2008162) following written informed consent from the patients.

### Method for final product preparation: ASCs in Cytocare® 532

Human SVF was isolated from lipoaspirate obtained from three randomly chosen healthy volunteers undergoing optimized liposuction [38], using 3 mm cannulae. Briefly, adipose tissue was digested with collagenase (0.120U/ml, Roche, Indianapolis, IN, USA) at 37 °C for 30 min and under constant shaking. Digestion was stopped by adding Dulbecco’s Modified Eagle’s Medium (DMEM with glutamax, Gibco (Invitrogen, Carlsbad, CA, USA) containing 10 % fetal calf serum (FCS, HyClone, Logan, UT, USA). Floating adipocytes were discarded and cells from the SVF were pelleted, rinsed with medium, centrifuged (300 g for 5 min. at 20 °C) and incubated in an erythrocyte lysis buffer for 20 min at 37 °C. This cell suspension was centrifuged (300 g for 5 min, 20 °C) and cells were counted using the Trypan blue exclusion method.

A total of 40,000 SVF cells/cm^2^ were plated and grown in proliferation medium containing DMEM (Gibco, Life technologies), HAM-F12 L-Glutamine (Gibco®, Life technologies, St Aubin, France) (v/v), 10 % FCS (HyClone), 10 ng/ml basic fibroblast growth factor (FGF2, Miltenyi Biotec, Paris, France), 10 μg/ml of gentamicin and 100 UI/ml penicillin (Panpharma, Fougeres, France). The medium was changed three times a week until 80 % confluence was reached. At subconfluency, cells were detached with trypsin-0.01 % EDTA (Invitrogen) and centrifuged for 10 min at 300 g and amplified in subculture at 4,000 cells/cm^2^ density (passage 1). At subconfluency, the cells were trypsinized, counted and cryopreserved in FCS/DMSO (90/10, v/v) (DMSO, Wak Chemie Medical GmbH, Steinbach, Germany). Samples were harvested for all the quality controls described below. On the day of surgery, the cells were thawed, washed, counted, and then mixed with the vehicle (Cytocare® 532) for injection into nude mice at a density of 1.10^6^ cells/ml of Cytocare® 532.

### Characterization of adipose-derived stem cells

Unless indicated, all chemicals were purchased from Sigma Aldrich, St. Louis, MO.

Cells were characterized for their phenotype using flow cytometry. Briefly, after detachment, cells were re-suspended in PBS at a concentration of 1-2 million/mL. Then, 100 μL of this cell suspension were stained in PBS using FITC-coupled CD45 and PE-coupled CD90, CD73, CD14, CD34, and HLA-DR antibodies or appropriate isotypic controls (All from BD Biosciences, Le pont de Claix, France). At least twenty thousand events were acquired with a FACSCanto II cytometer (BD Biosciences, Le Pont de Claix, France) and analyzed (DIVA software).

The cells were then tested for their ability to differentiate into various mesodermal lineages. For adipogenic differentiation, confluent cells at passage 1 were induced using adipogenic medium consisting of DMEM supplemented with 10 % of FCS, 10 μg/mL of 3-isobutyl-1-methylxanthine (IBMX), 100 μM of indomethacin, 1 μM of dexamethasone and 200 mUI of insulin (Umulin, Lilly laboratories, Neuilly-sur-Seine, France). After 14 days, lipid droplets were stained using 0.4 % Oil Red O solution after 10 % formalin fixation.

For osteogenic differentiation, sub-confluent cells at passage 1 were induced using a StemPro® osteogenesis differentiation kit (Gibco®, Life technologies, St Aubin, France). After three weeks, cells were fixed and stained with 40 mM Alizarin red (Merck Millipore, Fontenay sous Bois, France) to visualize calcium deposition.

Chondrogenic differentiation was evaluated using the high-density pellet culture approach, as previously described [39]. Briefly, subconfluent cells at P1 were detached using trypsin-EDTA, numerated, and 2.5×10^5^ viable ASCs were centrifuged (300 g, 10 min, two times) in a V-bottom 96-well plate (BD Biosciences, Le Pont de Claix, France) to form a pellet. The pellets were treated for 28 days with defined chondrogenic medium, which consisted of DMEM-F12 (4.5 g/L glucose, Life Technologies, St Aubin, France), 1 % of insulin-transferrin-selenium (ITS, Life Technologies, St Aubin, France), 100 nM dexamethasone, 170 μM L-ascorbic acid-2-phosphate, 1 mM sodium pyruvate, 350 μM L-proline, 10 ng/mL of transforming growth factor β3 (TGFβ3, R&D Systems, Minneapolis, MN, USA) and 50 ng/mL bone morphogenetic protein-2 (BMP-2, InductOs, Wyeth, Taplow, UK).

Proliferation medium was used for each differentiation control condition. The medium was changed three times a week.

### In vivo experimental protocol in nude mice

The present study was performed in accordance with the *Guide for the Care and Use of Laboratory Animals*, published by the National Institutes of Health (NIH Publication No. 85–23, revised 1996). All the experimental procedures were approved by the Ethics Committee of Lyon I Claude Bernard University (DR-2014-25).

Thirty male, nude, seven-week-old mice were anesthetized with buprenorphine (0.1 mg/kg) and ketamine (20 mg/ml) and surgery was performed under standard sterile conditions. One circular, full thickness 12 mm diameter wound was created on the back of each mouse and a ring of silicone (Folioxane®, ref FU050M, Novatech, La Ciotat, France) was sutured in place to prevent skin retraction.

During the follow-up, buprenorphine was administrated intra-peritoneally every day at a dose of 0.1 mg/kg/day during the first week and 0.05 mg/kg/day during the second week to avoid suffering. Each wound on each mouse was injected intradermally with 1 mL of final product containing 1.10^6^ ASCs (500 μL Cytocare 532 containing 0.5 × 10^6^ cells) around the wound into four injection sites and the other 500 μL applied onto the wound surface as previously described [20].

Briefly, one wound was created on each of the 30 mice randomly divided into three groups: 6 mice in the spontaneous healing (SH) group, 12 mice in the vehicle group (Cytocare® 532), and 12 mice (four per each ASC donor) in the ASCs group (ASCs in Cytocare® 532).

### Tolerance evaluation

Tolerance was assessed by the detection of serious side-effects (death), observation of the state of the wounds (oozing, infection), and general monitoring of the animals through weight gain and finally pain, using the McGill pain scale [40].

### Wound closure measurements

Every day, mice were observed and digital images were taken. Wound area was measured by tracing the wound margin and calculating the pixel area using image analysis software (Adobe Photoshop 7.0).

The wound healing rate was calculated as follows:

$$ 100-\frac{\left(\mathrm{Surface}\ \mathrm{o}\mathrm{f}\ \mathrm{actual}\ \mathrm{n}\mathrm{o}\mathrm{n}\ \mathrm{r}\mathrm{e}\hbox{-} \mathrm{epithelialized}\ \mathrm{zone}\right)}{\left(\mathrm{Surface}\ \mathrm{at}\ \mathrm{D}0\right)}\times \kern0.62em 100 $$

### Histological and immunohistochemical analyses

On day 27, the scar tissue was harvested with a rim of healthy normal tissue. Tissue samples were fixed in 10 % buffered formalin before embedding in paraffin. For histological analysis, tissue sections (3 μm) were deparaffinized, rehydrated and then stained with Masson’s trichrome or picro-sirius red. Following staining, slides were dehydrated with graded solutions of ethanol and methylcyclohexane baths and sealed with Permount mounting medium (Thermo Fisher Scientific, Saint Aubin, France).

For Masson’s trichrome, stained slides were scanned with a Mirax Scanner (Zeiss, Marly le Roi, France) and images were taken using a digital camera (Hitachi HV F22) and Mirax Scan 150 software (v 1.2, Zeiss). Images were analyzed using Pannoramic viewer software (v 1.15, 3DHistech, Budapest, Hungary). For picro-sirius red, stained slides were observed under polarized light (Leica DM 2000) and images were taken using a digital camera (Leica DFC420C) and LAS v4 software (Leica).

For immunohistochemical examination, all the washing steps consisted of three times 5 min each in PBS containing 0.2 % Tween 20. Three μm-thick sections were deparaffinized and rehydrated using methylcyclohexane, graded ethanol and running tap water baths. Antigens were retrieved using ficin for 10 min at 42 °C (Digest-All 1 Ficin, Life Technologies, St Aubin, France) and sections were then cut. Endogenous peroxidase activity was inactivated for 20 min with PBS containing 3 % normal goat serum (NGS, Merck Millipore, Fontenay sous Bois, France) and 5 % hydrogen peroxide (Sigma Aldrich, Saint-Quentin Fallavier, France). Afterwards, non-specific protein binding was avoided by blocking with PBS containing 5 % NGS. Primary mouse anti-mouse alpha smooth muscle actin (clone αsm-1, Leica Microsystems, Nanterre, France) was diluted 1:200 in PBS containing 5 % NGS and applied to the sections that were incubated overnight at 4 °C in a humid chamber. The sections were then washed and the secondary antibody (Envision + system HRP labelled polymer anti-mouse, Dako, Les Ulis, France) was applied for 45 min at room temperature. After a last washing step, the detected antigens were visualized by incubation with 3,3′-diaminobenzidine (Liquid DAB+ Substrate Chromogen System, Dako, Les Ulis, France). Finally, sections were counterstained with Mayers hematoxylin, and mounted using Faramount aqueous mounting medium (Dako).

### Assessment of skin perfusion

For all the experiments, animals were anesthetized with isoflurane and then placed in a heated environment to maintain a stable cutaneous temperature (35.0 ± 0.5 °C) throughout the measurements. Mice were placed in the prone position and skin blood flow (SBF) was measured using a laser Doppler imager (PERISCAN PIM3, Perimed, Sweden).

SBF was recorded for 1 min and signal analysis was performed off-line by calculating the average perfusion on each surface (43 mm^2^) of interest (PIMSoft software, Perimed, Järfälla, Sweden).

### Assessment of cutaneous microcirculation

To test the cutaneous microcirculation, two experiments were conducted in the three groups as previously described [41, 42]: endothelium-independent vasodilation in response to sodium nitroprusside (SNP) was evaluated, as well as endothelium-dependent vasodilation in response to acetylcholine (ACh).

For the experiments, animals were anesthetized by intraperitoneal injection of sodium thiopental (65 mg/kg). The level of anesthesia was determined by testing eye reflexes and tail pinch. The animals were then settled in an incubator (MMS, Chelles, France) heated to maintain a stable cutaneous temperature (35.0 ± 0.5 °C). Mice were placed in the prone position, followed by a 20 min resting period to stabilize the blood pressure and cutaneous temperature. Tail noninvasive blood pressure (IITC, Woodland Hills, CA) was recorded before and after the experiments to verify mean arterial blood pressure (MABP) stability. At the end of each experiment, the animals were killed by an overdose of thiopental.

To test endothelium-independent and -dependent responses, skin blood flow (SBF) was recorded during transcutaneous iontophoresis, using a laser Doppler multifiber probe (481-1, Perimed) applied to a 1.2 cm^2^ hairless area on the back of the animals. Endothelium-independent response was assessed using cathodal SNP iontophoretic delivery (67 mmol/L; Nitriate^®^; SERB, Paris, France) with the application of a 100 μA current for 20 s. Endothelium-dependent response was assessed using anodal ACh iontophoretic delivery (5.5 mmol/L; Sigma, Saint Quentin Fallavier, France) with the application of a 100 μA current for 10 s. SNP and ACh were dissolved in deionized water. SNP- and ACh-induced vasodilator responses are reported as the maximal percentage increase from baseline in response to the iontophoretic delivery of SNP and ACh, respectively.

### Statistical analysis

Statistical analysis was performed using Graphpad Prism 4 software. All the data are expressed as means ± SEM. For the wound healing rate, unpaired t-tests were carried out to evaluate statistical significance. For complete wound closure, the Kruskal-Wallis test was followed by a Dunn’s multiple comparison test to estimate the significance of differences for between-group comparisons. For skin perfusion and assessment of endothelium-independent and -dependent response experiments, one-way analysis of variance (ANOVA) was followed by a multiple comparison test to estimate the significance of differences for between-group comparisons. Significance was defined at *p* < 0.05.

## Results

### ASC characterization

**Fig. 1:**
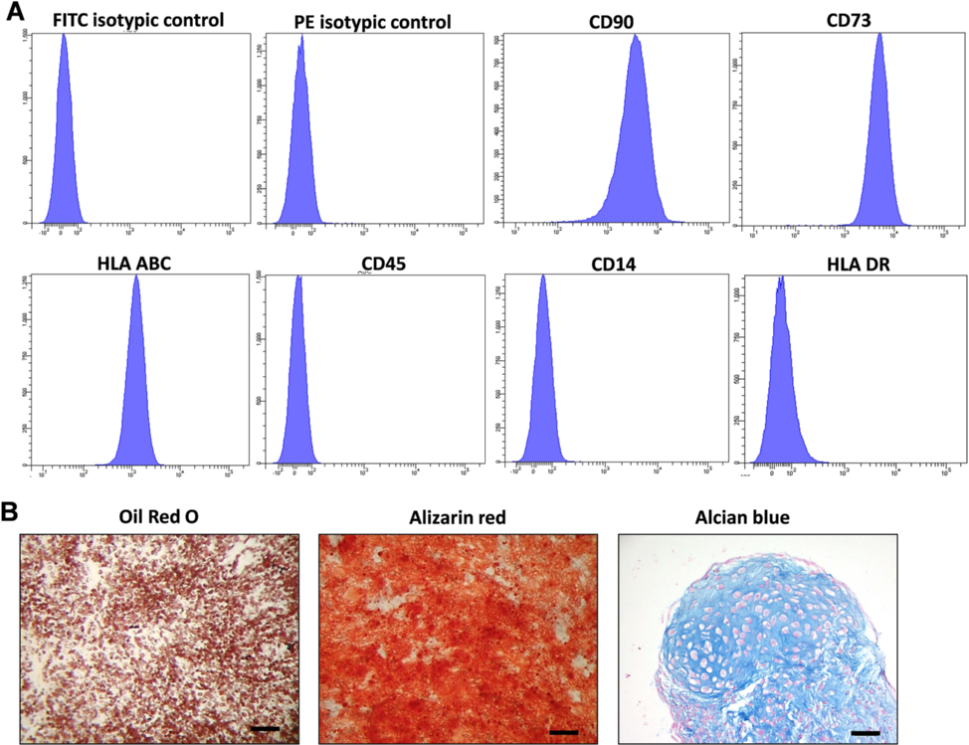
Immunophenotyping and trilineage differentiation of ASC. **a** ASCs were stained with antibodies and analyzed by flow cytometry. ASCs expressed markers CD73, CD90, and HLA ABC and were negative for CD45, CD14, and HLA DR. Representative histograms are shown. **b** ASCs were cultured in adipogenic, osteogenic or chondrogenic differentiation medium. Cells were fixed and stained with Oil Red O for adipogenesis, Alizarin red for osteogenesis and Alcian blue for chondrogenesis. Representative images are shown. Scale bar = 100 μm. *ASCs* adipose-derived stem cells, *CD* cluster of differentiation, *HLA* human leukocyte antigen

Adipose-derived stem cells (ASCs) were isolated using our laboratory’s routine method. Briefly, adipose tissue was collagenase-digested and cells were collected as the stromal vascular fraction (SVF) and grown in proliferation medium until passage 1 (two subcultures). Cells were characterized by flow cytometry and displayed a mesenchymal stem cell phenotype (Fig. 1a) as more than 98 % of the cells expressed CD90, CD73, and HLA-ABC and less than 2 % expressed CD14, HLA-DR, and CD45. Furthermore, these cells were able to differentiate towards adipogenic, osteogenic, and chondrogenic lineages, as shown by Oil red O, Alizarin red, and Alcian blue staining, respectively (Fig. 1b).

### Animal experiments

Throughout the experiment we had to exclude three mice for the loss of the ring causing a strong retraction of the wound and skewing the results except for tolerance. Analysis of the effectiveness was performed on 27 wounds: 6 untreated SH controls, 11 with the vehicle alone (Cytocare® 532), 10 with ASCs in Cytocare® 532.

### Tolerance

No serious side effects were observed following treatment. Whatever the treatment, no secondary infection was identified after the wounds. Only normal exudation phases were observed for each mouse, more or less late in the study. No behavioral abnormalities were detected in the mice with regard to food intake and spontaneous motor activity. An average 10 % weight gain was observed during the study period.

### Effects of ASCs on the kinetics of skin healing

**Fig. 2:**
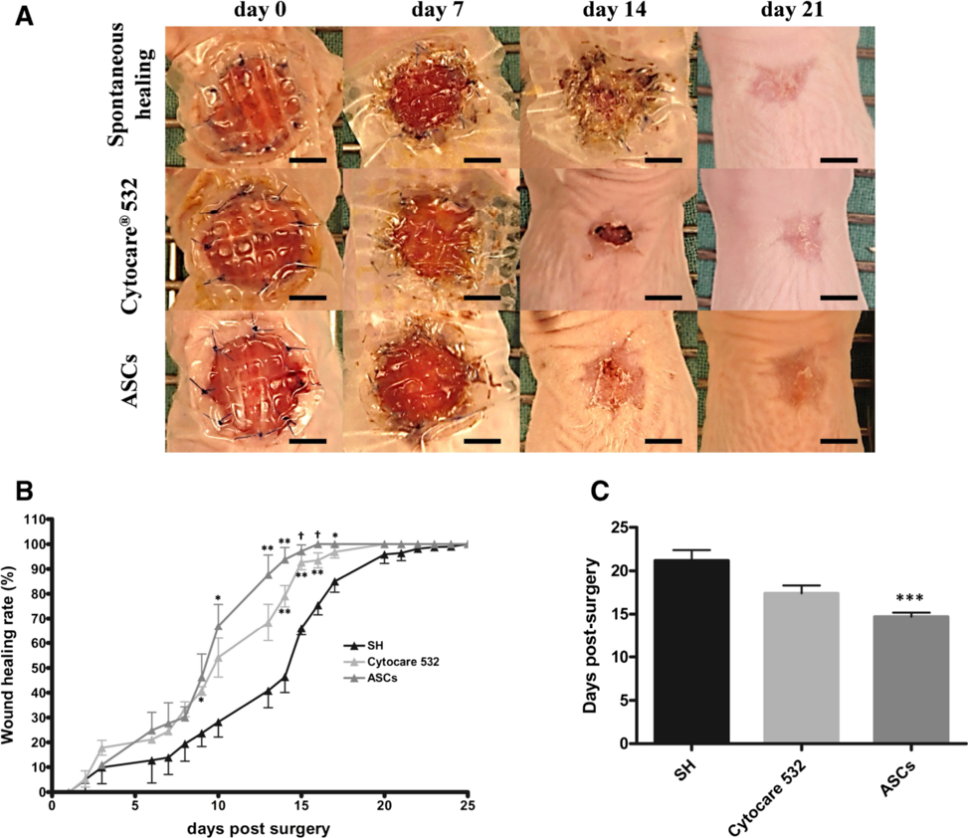
Influence of ASCs on wound healing kinetics. **a** After surgery, nude mice were intradermally injected or not with Cytocare® 532 or ASCs. Representative images are shown for 0, 7, 14, and 21 days post-surgery. Scale bar 5 mm. **b** Percentage of wound healing was monitored every day until complete wound closure. Results are expressed as means, with error bars indicating SEM. Statistical analysis was performed using the unpaired *t*-test. * *p* < 0.05, **p < 0.01 and † *p* < 0.001 compared to spontaneous healing. **c** Days to complete wound closure were noted. Results are expressed as means of days needed to reach complete closure and error bars indicate SEM. The Kruskal-Wallis test was followed by Dunn’s multiple comparison tests to estimate the significance of differences for between-group comparisons. *** *p* < 0.001 compared to spontaneous healing. *ASCs* adipose-derived stem cells

All wounds had an initial area of around 110 mm^2^ (radius = 12 mm). Figure 2a shows the representative digital images of the evolution of wound healing for each group at 0, 7, 14, and 21 days post-surgery. Figure 2b shows the percentage of wound healing rate over time post-surgery. During the first week, no differences were observed between the different treatments: 20 % of the wound was re-epithelialized. Then, from the seventh day post-surgery, a significant acceleration of wound healing was observed, for all treatments, compared to untreated wounds (SH group). By the thirteenth day, there was 85 % epithelialization with cultured ASCs, 68 % in the vehicle group and 40 % without treatment.

The time to complete healing was significantly shortened after treatment with ASCs (Fig. 2c). Indeed, in the group treated with ASCs, the number of days to closure was significantly lower than in the two control groups, 14.6 ± 0.3 days for ASC in Cytocare^®^ 532, 21.2 ± 1.1 (p < 0.001) days without treatment, and 17.4 days ± 0.8 with Cytocare® 532 alone, p <0.05. Thus, these results show that treatment with ASCs represents a gain of time to wound closure of more than six days versus SH and 2.8 days versus vehicle alone.

### Histological analysis of scar tissue

**Fig. 3:**
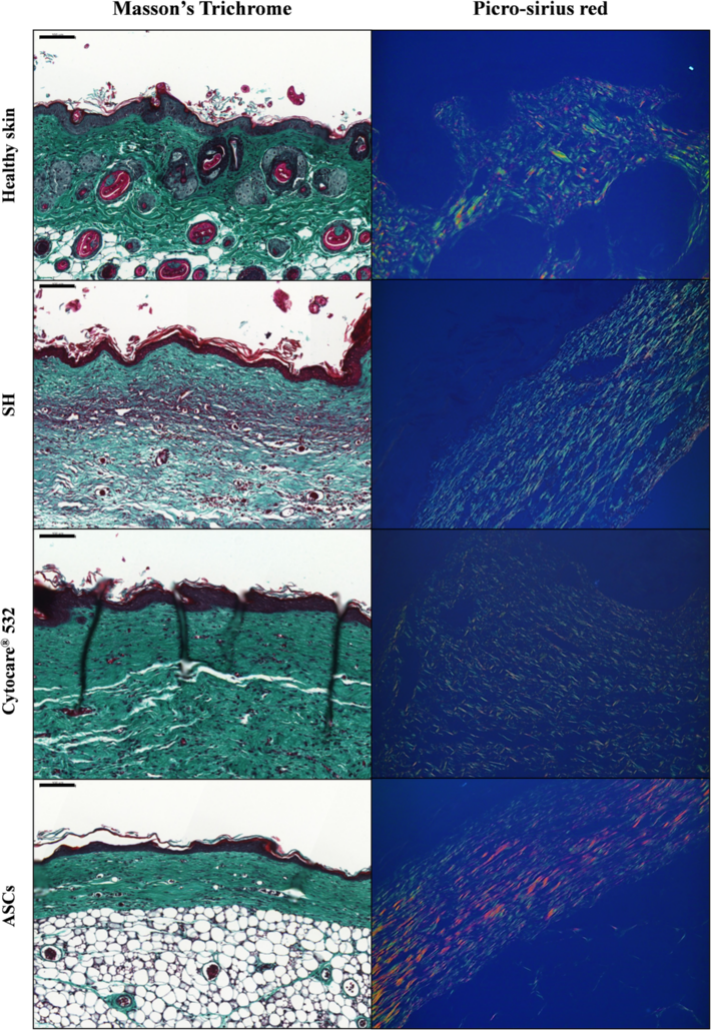
Wound histology after Masson’s trichrome and Picro Sirius red staining. Tissue sections obtained from the wound area at day 27 after cell injection were stained with Masson’s trichrome (*left panel*) and Picro Sirius red (*right panel*). Representative micrographs of wound histological images are shown. *ASCs* adipose-derived stem cells

Masson’ trichrome staining (Fig. 3) was used to analyze the overall quality of scarred areas. All the biopsies presented a good stratified and differentiated epithelium. The dermis appeared much thinner in the ASC group, with more homogenous ECM than in the two controls groups, SH and Cytocare® 532, in which the dermis appeared less vascularized and more inflammatory. Moreover, picro-sirius red staining under polarized light showed more red collagen fibers in the ASC group than in the two control groups.

### Skin perfusion

Skin perfusion, measured on the scar tissue on day 27, was significantly higher in the ASCs group (ASCs 4.4 ± 0.5 a.u./mm than in the untreated (2.5 ± 0.4 a.u./mm^2^) and vehicle 2.8 ± 0.2 a.u./mm^2^; *P* < 0.01) groups. There was no statistical difference between the untreated and vehicle groups.

### Assessment of endothelium-dependent response

In the untreated and vehicle groups, skin blood flow (SBF) increased in response to iontophoretic delivery of ACh, corresponding to an endothelium-dependent vasodilation of 33 ± 6 % and 33 ± 3 %, respectively. The ACh-induced vasodilation observed in the ASCs group (55 ± 7 %) was significantly higher than in both the untreated and vehicle groups (*P* < 0.05).

### Assessment of endothelium-independent response

In all groups, SBF increased in response to iontophoretic delivery of sodium nitroprusside (SNP) and no difference was observed in the endothelium-independent vasodilation between groups (untreated 47 ± 13 %, vehicle 51 ± 6 %, ASCs 44 ± 8 %).

### Quantification of dermal capillary density

**Fig. 4:**
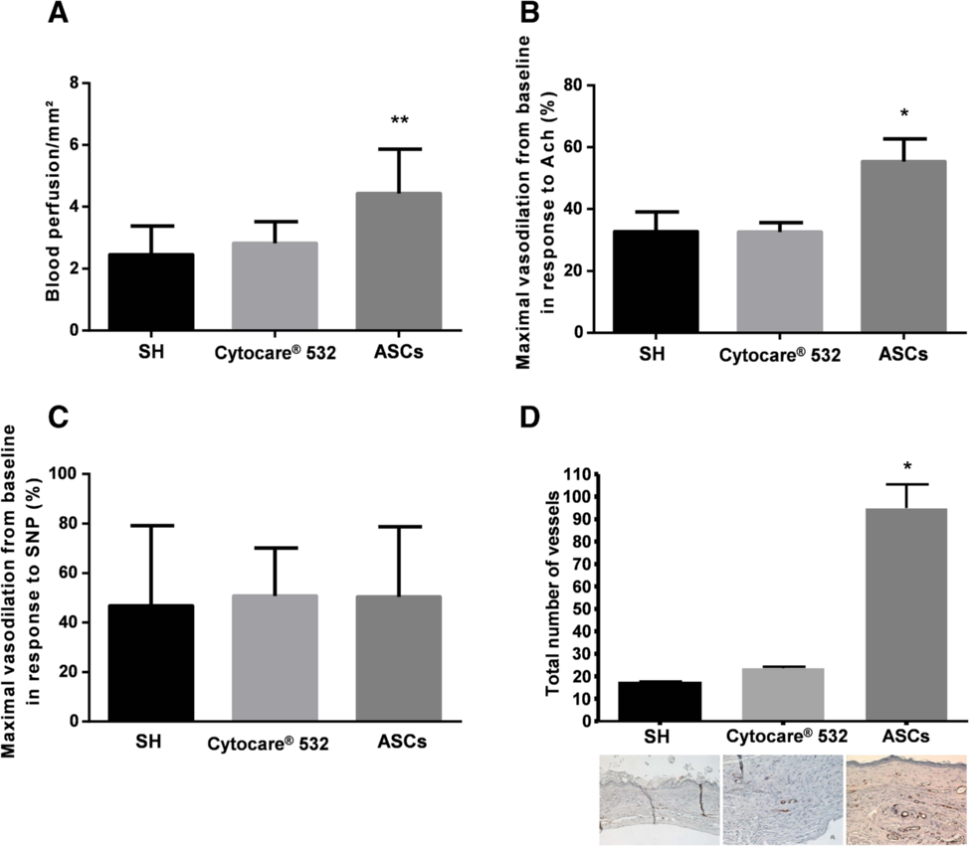
Quantitative and functional aspects of blood perfusion in the healed tissue. **a** Quantification of perfusion at day 27 after surgery was performed on the healed tissue compared with normal skin using laser Doppler imaging. **b** Percentage of vasodilation after ACh iontophoresis and **c** after SNP iontophoresis in SH-, Cytocare® 532- and ASC-treated mice. **d** One-way ANOVA was followed by a multiple comparison test to estimate the significance of differences for between-group comparisons. Significance was defined at *P* < 0.05 and ** *p* < 0.01 vs. control. Tissue sections obtained from the wound area 27 days after cell injection were stained with antibody against alpha smooth muscle actin and blood vessels in the total wound area were quantified. Representative images of the staining are displayed for each group. The Kruskal-Wallis test was followed by Dunn’s multiple comparison test to estimate the significance of differences for between-group comparisons. * *p* < 0.05 compared to spontaneous healing. All data are expressed as mean ± SEM. *Ach* acetylcholine, *ASCs* adipose-derived stem cells, *SH* spontaneous healing, *SNP* sodium nitroprusside

Immunohistochemistry of αSMA (Fig. 4d) showed no significant differences between the two control groups, as the number of total blood vessels in the healed area was 17.33 ± 0.27 and 23.67 ± 0.54 for the SH and Cytocare® 532 groups, respectively (p > 0.05). Although there was no statistical difference in the vascular density between ASC-treated and Cytocare 532-treated mice, the ASCs in Cytocare 532 treatment showed an increase in the number of blood vessels to 95 ± 9.3.

## Discussion

Many treatments using SVF [43, 44] and adipose-derived stem cells (ASCs) are already in use [45–48] in plastic and cosmetic surgery. The main problem encountered with SVF-based therapy is the poor reproducibility of the outcomes, which might be due to the insufficiency of ASCs. For this reason, SVF is now often used in combination with fat in what is known as cell-assisted lipotransfer (CAL) [45, 49, 50].

In this preclinical study, we demonstrate the tolerance and therapeutic effectiveness of human cultured ASCs in a mouse wound model. The cells were intradermally administered in a skin-protecting and antioxidant nutrient vehicle, Cytocare® 532, for which the biocompatibility and bioavailability of ASCs in Cytocare® 532 were previously shown [37]. In terms of in vivo tolerance, no impact was noticed on mortality and no infection was observed despite the absence of antibiotics. In all the tested groups, the pain due to the excisional wound, evaluated on the Mogill pain scale [40], was controlled by the daily administration of buprenorphine over the first two weeks.

Evaluation of the therapeutic efficacy of ASCs must take into account the characteristics of the chosen wound model in our study. In this model, a Galiano silicone ring was sutured to the edges of the wound to prevent retraction and lengthen the average healing time by eight days, as previously shown [20, 51, 52]. It is now considered that the average time for the complete closure of a 10 mm wound increases from 12 days without, to 20 days with, a ring [23]. Our results were essentially the same in the SH group with 21 days for a slightly larger wound (12 mm), and with an accurate assessment since the readings were performed frequently. The use of a transparent dressing greatly improves wound tracking from both an ethical and a practical point of view as it permits daily monitoring of the percentage of epithelialization and the time to complete wound closure without the need to sacrifice the animal before each observation, as has been done in some experimental protocols [25]. In the literature, the assessment of wound closure is usually performed once or twice per week [23, 53], or sometimes only once during the entire healing period [54], by removing the dressing after the mice are under anesthesia, with the additional risk of tearing the newly formed epithelium, or sacrificing an animal at each observation.

Overall, when considering the main evaluation criterion, i.e., the time for full wound closure, the improvements we have made to the Galiano model include: 1) a daily reading of re-epithelialization, made possible by the use of transparent dressings; and 2) increases in the initial wound surface (12 mm instead of the 6 mm often practiced) [20, 24]. This greater wound area will increase the intra-group reproducibility (in this study, the SEM for the mean time to wound closure ranged from 0.3 % to 1.1 depending on the group) and the sensitivity of the model to assess treatments promoting wound healing.

Using this model, the effect of ASCs on wound healing was shown, by significantly shortening the time to complete healing by more than six days, when compared to the SH group (p < 0.001). Overall, these results confirm the literature data showing accelerated healing after administration of ASCs [20, 53]. However, it should be noted that these studies differ from ours by the choice of model (see above) and the vehicle, which in some cases is also not specified. With no need to pull off dressings, the use of a vehicle [37, 55] to avoid dilution or loss of ASCs after injection, as well as the daily monitoring of healing, we put aside many biased interpretations and facilitated the experiment.

Our results also suggest that Cytocare® 532, selected as the vehicle, operates to potentiate the ASC effect. In fact, its main component, hyaluronic acid [56], a macromolecule forming a fundamental part of the extracellular matrix of the skin, plays a vital role in the healing process by controlling cell migration and proliferation, as well as modulating the inflammatory and angiogenic processes in the early stages of healing, and progressive tissue remodeling [57–60].

Beyond the healing time criterion, one of the therapy objectives now being tested in healing models is obtaining a good repair, the closest possible to healthy skin to prevent recurrence of the wound and the development of a chronic wound, as often observed in obese and diabetic patients. Histological aspects of the skin revealed by Masson’s trichrome staining showed that the treatment strongly improved the quality of the dermis which seemed to be in the remodeling phase in ASC-treated mice, while still in the proliferative phase with many inflammatory cells in the SH and Cytocare® 532 groups (Fig. 3, left panel). Furthermore, picro-sirius red staining highlighted the presence of type I collagen, as shown by the presence of red fibers in the healed tissue, whereas the majority of the fibers observed in the SH and Cytocare® 532 groups were green, revealing the presence of type III collagen [61, 62].

One of the most important parameters for quality healing is vascularization. A perfusion defect, whether it is associated with poorly functioning blood vessels or low neo-angiogenic levels, leads to a risk of relapse, sustainability of the wound or necrosis. Our results show that there is no defect in vascular smooth muscle function (Fig. 4a, b and c), suggesting that the enhanced endothelial response observed in the ASC groups could reflect an improved endothelial function within the wound healed tissue under ASC treatment. An increase in dermal vascular density (Fig. 4d) could suggest that ASC treatment could promote angiogenesis during the wound healing process, which, in turn, could also lead to improved microcirculation and to a reduced wound healing delay, compared to the control groups. The potential of ASCs in promoting revascularization has already been shown [33].

These results suggest that in our wound model, ASCs can improve perfusion by directly affecting the function of these vessels or indirectly through a reduction in fibrosis and acceleration of the healing process. Overall, our study shows the therapeutic benefit of ASCs in the wound healing context and their effect on shortening the healing period, increasing perfusion. The healing effect that we observe here is promising for application, particularly in cases of chronic wounds that sometimes lead to a therapeutic impasse that could become life-threatening for some patients.

## Conclusions

The tolerance to, and efficacy of, cryopreserved ASCs, to rapidly obtain the complete closure of a wound by increasing the maturation of the skin and its blood perfusion *s*hows their therapeutic benefit in the wound healing context, reinforcing our in vitro results using a three-dimensional model demonstrating the potential of ASCs to obtain a complete stratified and differentiated epidermis [63].

## Abbreviations

Ach: Acetylcholine
ASC: Adipose-derived stem cells
BMP2: Bone morphogenetic protein 2
CD: Cluster of differentiation
DAB: 3,3′-Diaminobenzidine
DMEM: Dulbecco’s modified Eagle’s medium
DMSO: Dimethyl sulfoxide
EC: European Community
EDTA: Ethylenediaminetetraacetic acid
FCS: Fetal calf serum
FGF: Fibroblast growth factor
FITC: Fluorescein isothiocyanate
HA: Hyaluronic acid
HLA: Human leukocyte antigen
HRP: Horseradish peroxidase
IBMX: 3-Isobutyl-1-methylxanthine
ITS: Insulin-transferrin-selenium
LDF: Laser Doppler flowmetry
LDI: Laser Doppler imaging
MABP: Mean arterial blood pressure
NGS: Normal goat serum
PBS: Phosphate buffered saline
SBF: Skin blood flow
SEM: Standard error of the mean
SH: Spontaneous healing
SNP: Sodium nitroprusside
SVF: Stromal vascular fraction
TGFβ3: Transforming growth factor β3
